# Validation of *Plasmodium vivax* centromere and promoter activities using *Plasmodium yoelii*

**DOI:** 10.1371/journal.pone.0226884

**Published:** 2019-12-20

**Authors:** Kittisak Thawnashom, Miho Kaneko, Phonepadith Xangsayarath, Nattawat Chaiyawong, Kazuhide Yahata, Masahito Asada, John H. Adams, Osamu Kaneko

**Affiliations:** 1 Department of Protozoology, Institute of Tropical Medicine (NEKKEN), Nagasaki University, Sakamoto, Nagasaki, Japan; 2 Department of Medical Technology, Faculty of Allied Health Sciences, Naresuan University, Mueang, Phitsanulok, Thailand; 3 Leading Program, Graduate School of Biomedical Sciences, Nagasaki University, Sakamoto, Nagasaki, Japan; 4 Center for Global Health and Infectious Diseases Research, College of Public Health, University of South Florida, Tampa, Florida, United States of America; Instituto Rene Rachou, BRAZIL

## Abstract

*Plasmodium vivax* is the leading cause of malaria outside Africa and represents a significant health and economic burden on affected countries. A major obstacle for *P*. *vivax* eradication is the dormant hypnozoite liver stage that causes relapse infections and the limited antimalarial drugs that clear this stage. Advances in studying the hypnozoite and other unique biological aspects of this parasite are hampered by the lack of a continuous *in vitro* laboratory culture system and poor availability of molecular tools for genetic manipulation. In this study, we aim to develop molecular tools that can be used for genetic manipulation of *P*. *vivax*. A putative *P*. *vivax* centromere sequence (PvCEN) was cloned and episomal centromere based plasmids expressing a GFP marker were constructed. Centromere activity was evaluated using a rodent malaria parasite *Plasmodium yoelii*. A plasmid carrying PvCEN was stably maintained in asexual-stage parasites in the absence of drug pressure, and approximately 45% of the parasites retained the plasmid four weeks later. The same retention rate was observed in parasites possessing a native *P*. *yoelii* centromere (PyCEN)-based control plasmid. The segregation efficiency of the plasmid per nuclear division was > 99% in PvCEN parasites, compared to ~90% in a control parasite harboring a plasmid without a centromere. In addition, we observed a clear GFP signal in both oocysts and salivary gland sporozoites isolated from mosquitoes. In blood-stage parasites after liver stage development, GFP positivity in PvCEN parasites was comparable to control PyCEN parasites. Thus, PvCEN plasmids were maintained throughout the parasite life cycle. We also validated several *P*. *vivax* promoter activities and showed that *hsp70 promoter* (~1 kb) was active throughout the parasite life cycle. This is the first data for the functional characterization of a *P*. *vivax* centromere that can be used in future *P*. *vivax* biological research.

## Introduction

Malaria mortality and morbidity has been significantly reduced during recent decades through extensive control efforts but remains an important global health problem [1]. *Plasmodium vivax* malaria is difficult to control compared to *Plasmodium falciparum* due to its dormant hypnozoite stage in the host liver and its high transmission potential prior to the onset of clinical symptoms [2]. Hypnozoites are resistant to antimalarials targeting blood-stage parasites and additional drugs are required that are effective against liver stage parasites, such as primaquine or tafenoquine [3]. However, anti-relapse therapies present risks when administered to patients with glucose-6-phosphate dehydrogenase (G6PD) deficiency, since these drugs exhibit a potential life-threatening side effect of systemic hemolysis. In addition, primaquine-resistant vivax malaria has emerged [4] and variation in the cytochrome P450 2D6 metabolization of primaquine can significantly alter its efficacy [5–7]. These features make the control strategy for *P*. *vivax* more complicated than that for *P*. *falciparum*, and as a result the proportion of vivax malaria has been increasing [8].

Another unique feature of *P*. *vivax* is a reticulocyte preference for growth and development during the blood stages of infection. Due to the requirement for immature reticulocytes, and other unknown variables, no robust large-scale continuous *in vitro* cultivation method is available for *P*. *vivax*. As a result the biological features unique to *P*. *vivax* are poorly understood, such as the hypnozoite stage and reticulocyte preference, and drug discovery and therapy evaluations are hampered [9]. Therefore, *P*. *vivax*-specific research innovations are urgently needed. One such innovation would be establishing a transgenic reporter *P*. *vivax* line expressing fluorescent proteins and/or luciferase enzymes as a valuable tool to enhance the efficiency of *ex vivo* cultures or experimental animal infections.

To date, genetic elements to regulate protein expression are poorly characterized in *P*. *vivax*. Although promoters of *P*. *falciparum* histidine rich protein 3 (*hrp3*, ~2 kb) and calmodulin (*cam*, ~1 kb) were found to be active in *P*. *vivax* [10], only Barros et al. (2015) has successfully used a *P*. *vivax* promoter region, an ~1 kb fragment of *cam* (PVX_084825) to express a zinc-finger nuclease in *P*. *vivax* [11]. Several other promoters have been validated in the non-human primate malaria parasites *P*. *knowlesi* and *P*. *cynomolgi*, which are phylogenetically close to *P*. *vivax*, indicating that these promoters have a high potential to also be active in *P*. *vivax*. Promoters active in *P*. *knowlesi* are *P*. *falciparum hrp3* [12], *cam*, heat shock protein 86 (*hsp86*) [13], and chloroquine resistance transporter (*crt*) [14]; *P*. *berghei* dihydrofolate reductase/thymidylate synthase (*dhfr-ts)* [8], apical membrane protein 1 (*ama1*) [15], and elongation factor 1-alpha (*ef1-α*) [16]; and *P*. *chabaudi dhfr-ts* [17]. Promoters active in *P*. *cynomolgi* are *P*. *falciparum hrp3* and *P*. *berghei dhfr-ts* [18], and *P*. *knowlesi hsp70* and *ef1-α* [19]. The *P*. *cynomolgi* centromere region of chromosome 11 was also shown to be active [19], which is an important element to ensure efficient plasmid segregation to daughter cells.

Because a transfection system for *P*. *vivax* remains difficult to establish due to the need for parasite infections of non-human primates, it is important to have alternate approaches to experimentally validate regulatory elements that could be used for *P*. *vivax* transgene expression. Unfortunately, previous studies of *P*. *vivax* promoters (*msp1*, *dhfr-ts*, *vir3*, *vir23/24*, and *ef-1α*) found them to be inactive or poorly active in blood-stage *in vitro* cultures of *P*. *falciparum* [20]. Therefore, we decided to use a rodent malaria parasite *Plasmodium yoelii* as a platform, since many regulatory elements have been shown to be replaceable between rodent malaria parasites and old world monkey malaria parasites. In this study we evaluated *P*. *vivax* gene regulatory elements and centromere regions for activity in *P*. *yoelii* for potential suitability for genetic manipulation of *P*. *vivax*.

## Results and discussion

### Generation of plasmid constructs containing a *P*. *vivax* centromere region

**Fig 1:**
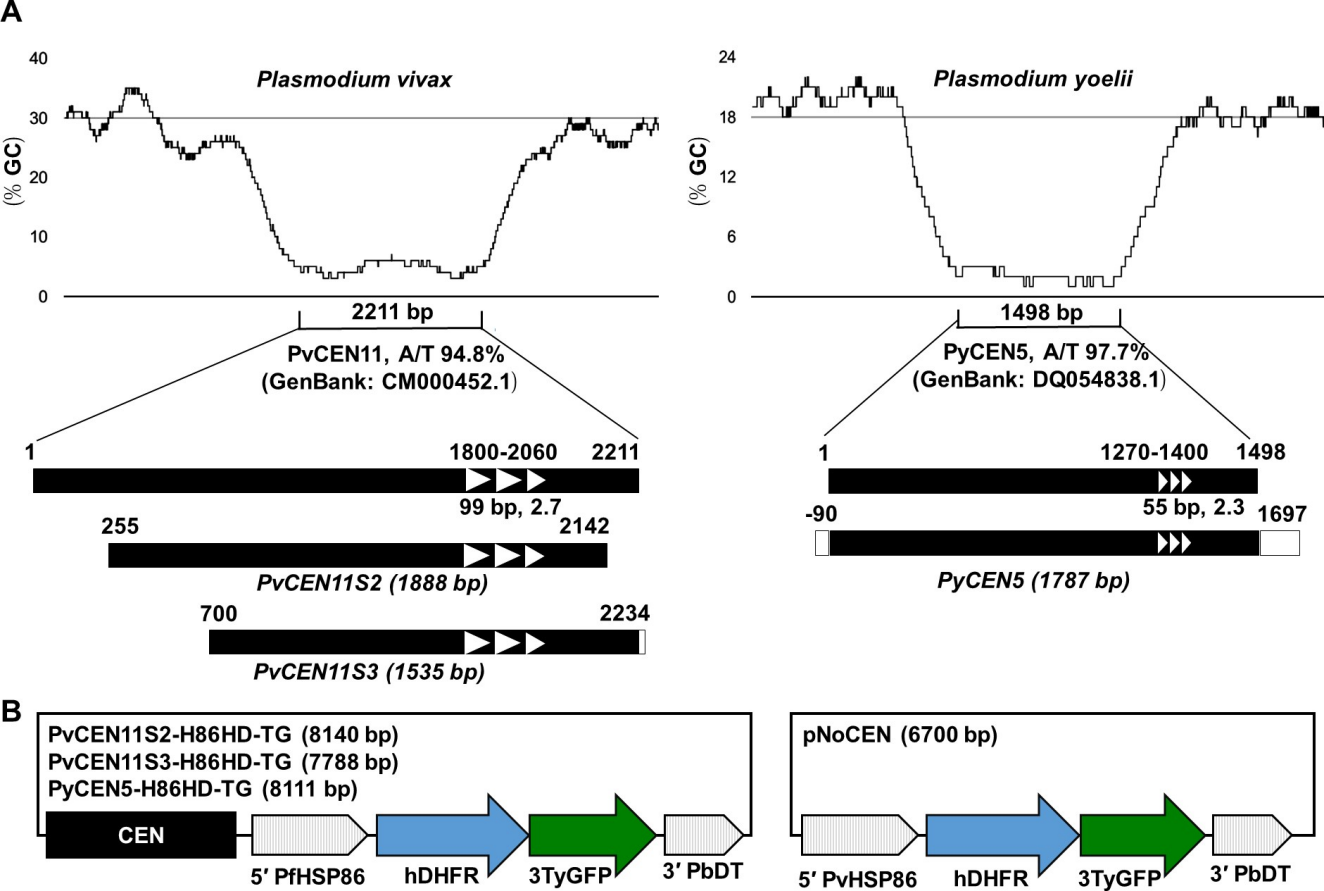
Schematic images of the centromere region of *P*. *vivax* and *P*. *yoelii* used in this study and the design of the plasmids to evaluate centromere function. **(A)** The *P*. *vivax* centromere sequence was selected from chromosome 11 (CM000452.1). DNA fragments of approximately 1.9 and 1.5 kb size were used for plasmid construction and named PvCEN11S2 and PvCEN11S3, respectively. The *P*. *yoelii* centromere sequence was selected from chromosome 5 (DQ054838.1) and approximately 1.8 kb DNA fragment was used for the plasmid, named PyCEN5. These sequences include repeat sequence motifs (white arrowhead). **(B)** Centromere plasmids evaluated in this study (PvCEN11S2-H86HD-TG, PvCEN11S3-H86HD-TG, and PyCEN5-H86HD-TG) and a control plasmid without a centromere region (pNoCEN). CEN, centromere region; 5' PfHSP86, the 5' untranslated region (UTR) of *P*. *falciparum* heat shock protein 86; hDHFR, human dihydrofolate reductase open reading frame; 3TyGFP, triple Ty1 tag and green fluorescent protein open reading frame; 3' PbDT, 3' UTR of *P*. *berghei* dihydrofolate reductase-thymidine kinase; and 5' PvHSP86, 5' UTR of *P*. *vivax* heat shock protein 86.

Putative *P*. *vivax* centromeres were predicted by gene synteny and whole genome sequence analysis for four putative centromeres identified in chromosomes 5, 9, 11, and 13 (Genbank numbers CM000446.1, CM000450.1, CM000452.1, and CM000454.1, respectively) [21–23]. We PCR-amplified and cloned these centromere regions (termed PvCEN5, PvCEN9, PvCEN 11, and PvCEN13, respectively) in *E*. *coli*. Plasmids containing the PvCEN5 and PvCEN9 fragments were unstable and were spontaneously deleted during the cloning procedure. Plasmids containing the PvCEN11 and PvCEN13 fragments were also unstable, but 10% of the transformed *E*. *coli* contained full-length fragments. After attempts to transfer these PvCEN fragments to a Gateway destination plasmid, we successfully generated a destination plasmid containing the PvCEN11 fragment (2211 bp). This fragment was then truncated in an attempt to make the PvCEN11-based plasmid more stable in *E*. *coli*; specifically, sequences 255–2142 (1888 bp) and 700–2234 (1535 bp) were selected to generate destination plasmids containing the fragments PvCEN11S2 or PvCEN11S3, respectively (Fig 1A). These sequences contained a highly AT-rich fragment and a repetitive region, which are likely required for centromere function [23].

Since there is no available *P*. *vivax* long-term *in vitro* culture, we used *P*. *yoelii* to evaluate if the putative centromeres were functional in retaining episomal plasmids during blood-stage parasite propagation. As a positive control, the predicted *P*. *yoelii* centromere region of 1787 bp in chromosome 5 (DQ054838.1, PyCEN5) was used to make a destination plasmid (Fig 1A). We then constructed three episomal centromere-based plasmids (PvCEN11S2-H86HD-TG, PvCEN11S3-H86HD-TG, and PyCEN5-H86HD-TG) expressing a drug selectable marker human dihydrofolate reductase (hDHFR) fused with a triple Ty1 tag sequence and a green fluorescence protein (GFP) marker driven by the *P*. *falciparum hsp86 (pfhsp86)* promoter (Fig 1B). A negative control plasmid containing the same hDHFR-3Ty-GFP expression cassette without a centromere region was also prepared.

**Fig 2:**
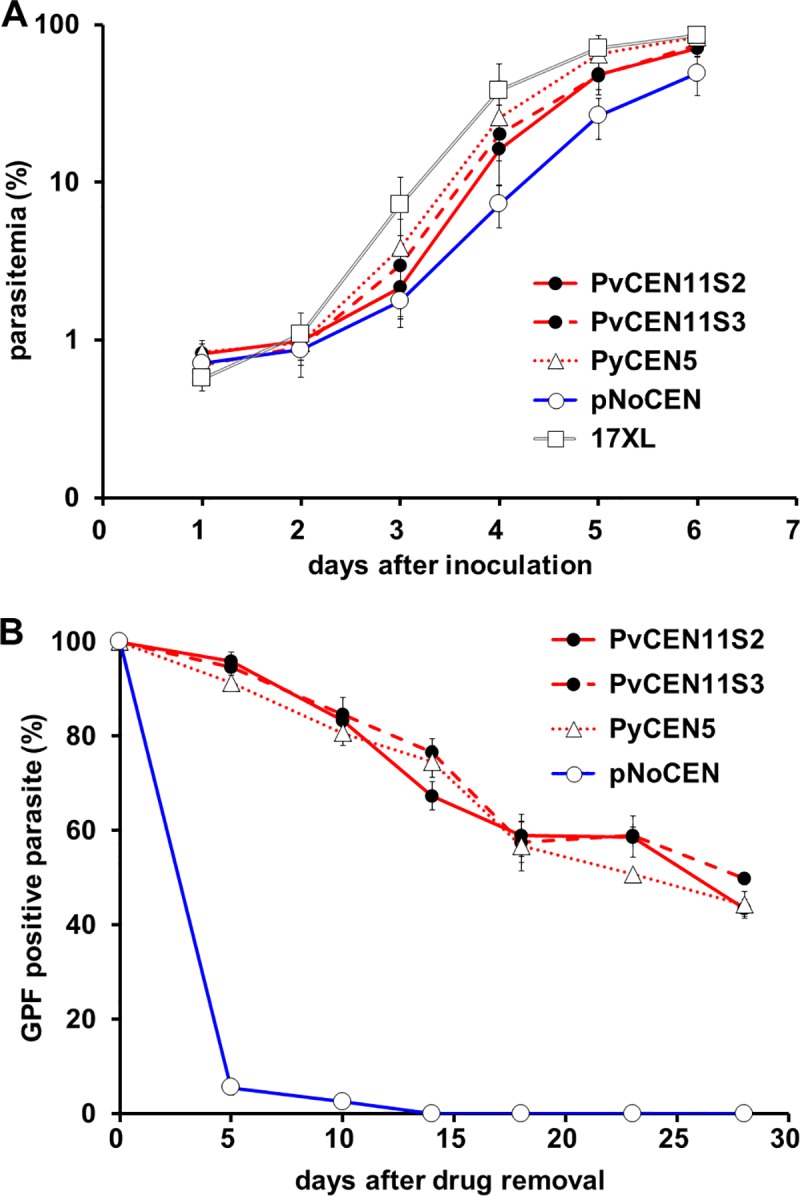
Growth curve and the percentages of GFP-positive parasites. **(A)** Infected red blood cells (1 x 10^6^) of transgenic parasites (PvCEN11S2, PvCEN11S2-H86HD-TG; PvCEN11S3, PvCEN11S3-H86HD-TG; PyCEN5, PyCEN5-H86HD-TG; and pNoCEN) or the parental parasite (17XL) were inoculated into each mouse and maintained with pyrimethamine except 17XL. Parasitemias were determined daily until day 7. **(B)** The percentage of GFP-positive parasites without pyrimethamine was examined at 7 time points until day 28. Mean and standard deviation from 5 mice were plotted for each group.

Transgenic lines were successfully generated with the 3 centromere-based plasmids using *P*. *yoelii* 17XL. However, transgenic parasites were not obtained with the negative control plasmid following multiple trials (two independent transfections and four times total). Thus, we decided to use the promoter region of *P*. *vivax hsp86* (*pvhsp86*), an ortholog of *Pfhsp86*, in the syntenic region for the negative control plasmid without any centromere region (pNoCEN), and a transgenic line was successfully obtained (Fig 1B). The reason for the failure to obtain a transfectant with the *Pfhsp86* promoter-based control plasmid is not clear, because the promoter activities in *P*. *yoelii* appear to be equivalent between the *Pfhsp86* and *Pvhsp86* promoters (described later). Of note is that the *Pfhsp86* promoter region does not appear to play a role in plasmid segregation to daughter cells [24], thus replacing this promoter is considered not to affect the plasmid segregation efficiency. Under pyrimethamine (PYR) drug pressure, 3 transgenic parasites containing centromere-based plasmids grew similarly to *P*. *yoelii* wild type 17XL line parasites maintained without PYR (Fig 2A). However, pNoCEN parasites grew slower with PYR than the 17XL line without PYR (Fig 2A). Although it was not significant, pNoCEN parasites showed a lower maximum growth rate per day compared to the other lines (S1A Fig). The number of merozoites per schizont were equivalent among all parasite lines, indicating that this is not the determinant of the observed slow growth phenotype of pNoCEN parasites (S1B Fig). We interpret that the slower growth rate of pNoCEN parasites is likely due to a low segregation efficiency of the pNoCEN plasmid to daughter merozoites.

### Plasmids containing *P*. *vivax* or *P*. *yoelii* centromere regions were stably and similarly maintained in the rodent malaria parasite *P*. *yoelii* during asexual growth *in vivo*

To evaluate the stability of the centromere-based plasmids during asexual growth, transgenic parasites were maintained without PYR pressure and the ratios of GFP-positive parasites were examined. Parasites were maintained in mice with PYR for one week to ensure that all parasites retained centromere plasmids, then infected red blood cells (iRBCs) were passaged to new mice and maintained without PYR for 28 days in naïve mice by syringe passage. When parasitemias reached 5–10%, blood was collected and 10^6^ iRBCs were inoculated intraperitoneally into a new mouse. We found that parasites with control pNoCEN rapidly lost a GFP signal and only 2.5 ± 0.8% and 0% GFP-positive parasites were observed on day 10 and 14, respectively. In contrast, the ratio of GFP-positive parasites gradually declined in transgenic parasites with PvCEN11S2, PvCEN11S3, or PyCEN5 with the percentage of GFP-positive parasites averaging 44 ± 2, 50 ± 1, and 44 ± 3% on day 28, respectively (Fig 2B). There was no significant difference in the percent infections among the three centromere-based plasmids (Fig 2B). Because the average number of merozoites per schizont was about 11 (= 2^3.46^), roughly 3.5 rounds of nuclear division was assumed to occur during one blood-stage schizogony of 18 hours [25]. Based on these assumptions, the segregation efficiency of the plasmid per nuclear division was calculated at each observation point. The segregation efficiency of parasites transfected with the PvCEN11S2 or PvCEN11S3 plasmids was always more than 99%, comparable with that of PyCEN5 parasites (> 99%), while the segregation efficiency of parasites transfected with a plasmid without a centromere, pNoCEN, was 88.3 ± 0.4% on day 5 and 92.2 ± 0.7% on day 10 (Table 1).

**Table 1:** Segregation efficiency at the end of multiplication periods.

Plasmid	Days after pyrimethamine removal					
	5	10	14	18	23	28
**PvCEN11S2**	99.8 (0.1)	99.6 (0.0)	99.4 (0.1)	99.4 (0.1)	99.5 (0.1)	99.4 (0.0)
**PvCEN11S3**	99.8 (0.1)	99.6 (0.1)	99.6 (0.1)	99.3 (0.1)	99.5 (0.0)	99.5 (0.0)
**PyCEN5**	99.6 (0.1)	99.5 (0.1)	99.5 (0.1)	99.3 (0.1)	99.4 (0.0)	99.4 (0.0)
**pNoCEN**	88.3 (0.4)	92.2 (0.7)				

The segregation efficiency of the plasmid per nuclear division was calculated (see Materials and methods) at each point of passage. Roughly 3.5 rounds of nuclear division occurring during one blood-stage schizogony of 18 hours was assumed [25], which resulted in the production of 11 daughter nuclei. Values are based on the parasitemia in 3 to 5 independent mice. Standard deviation is shown in parenthesis. Values were not calculated for pNoCEN after day 14, because no GFP-positive parasites were detected.

**Fig 3:**
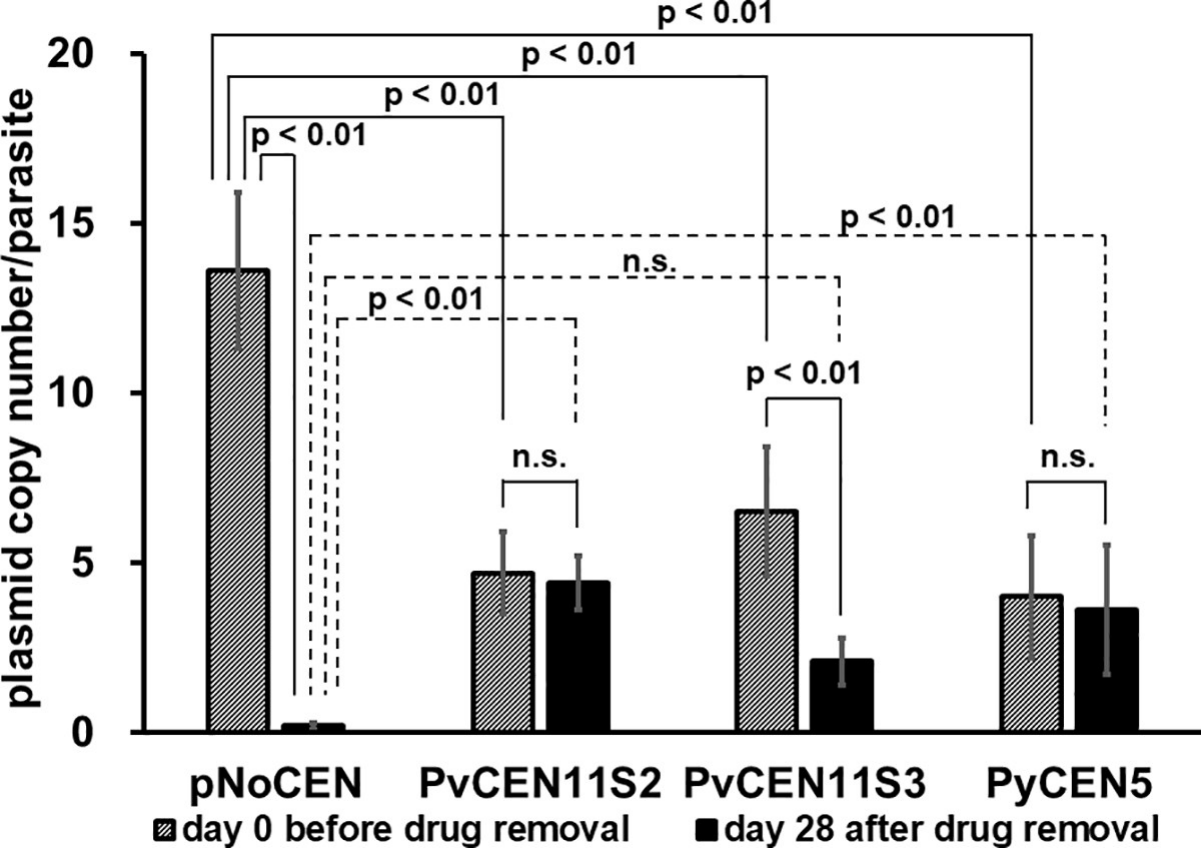
Change in plasmid copy numbers 28 days after removal of pyrimethamine. Plasmid copy numbers were quantitated before (day 0) and after (day 28) removal of pyrimethamine drug pressure in transgenic parasites with PvCEN11S2-H86HD-TG (PvCEN11S2), PvCEN11S3-H86HD-TG (PvCEN11S3), PyCEN5-H86HD-TG (PyCEN5), or pNoCEN. Three or five mice were used for each group on days 0 and 28, respectively. After the one-way ANOVA test indicated significant difference, Tukey's post-hoc multiple comparisons test was performed. Significant difference was indicated with p values. n.s. indicates not significant (p ≥ 0.05).

Next, we evaluated the plasmid copy number by qPCR before and 28 days after PYR removal. In the pNoCEN parasites, plasmid copy number was 14 ± 2 per parasite before PYR removal and 0.2 ± 0.1 per parasite on day 28 after PYR removal (~70-fold reduction, p < 0.01). The observation of a high copy number of pNoCEN is consistent with the concept that parasites with plasmid high copy numbers would be selected under drug pressure when the plasmid is not segregating efficiently. In the PvCEN11S2, PvCEN11S3, and PyCEN5 parasites, plasmid copy numbers were 4.7 ± 1.2, 6.5 ± 1.9, and 4.0 ± 1.8 per parasite before PYR removal and 4.4 ± 0.8, 2.1 ± 0.7, and 3.6 ± 1.9 per parasite on day 28 after PYR removal, respectively (Fig 3). These data indicate that the copy number of centromere-based plasmids is much lower than the copy number of pNoCEN with PYR (p < 0.01), but higher than that of pNoCEN without PYR (p < 0.01 for PvCEN11S2 and PyCEN5, but not significant for PvCEN11S3). Although the reduction in GFP positivity was similar among PvCEN11S2, PvCEN11S3, and PyCEN5 parasites, the plasmid copy numbers of PvCEN11S3 parasites were significantly reduced after 28 days cultivation without PYR (p < 0.01), albeit PvCEN11S2 and PyCEN5 parasites did not show significant reduction. The PvCEN11S2 445 bp sequence that is missing from PvCEN11S3 may have an important role in this observation.

Iwanaga et al (2010) showed that approximately 90% of *P*. *berghei* parasites with a plasmid containing the complete *P*. *berghei* centromere region (pbCEN5) expressed GFP on day 21 after PYR removal [26], significantly higher than the ~60% GFP positivity seen in this study in *P*. *yoelii* with PyCEN5 on day 18. This may be partly due to the difference in growth rates between the two parasite lines; *P*. *yoelii* 17XL line parasite invades a variety of RBCs and grows more rapidly than *P*. *berghei* which preferentially invades less abundant younger RBCs. The difference in the duration of one blood-stage schizogony may also contribute, which is 18 hours for *P*. *yoelii* [25] and 22–23 hours for *P*. *berghei* [27]. These are consistent with the observation that the segregation efficiency of the control pbGFPcon plasmid without a centromere region was 94% per nuclear division in their study, which was higher than the values for our control pNoCEN plasmid (88.3 or 92.2). Another factor may be that GFP was fused to hDHFR and expressed under one promoter in this study, whereas GFP and the drug selectable marker protein were expressed under different promoters for the *P*. *berghei* study. PvCEN11S2 and PyCEN5 parasites retained similar plasmid copy numbers per parasite before and on day 28 after PYR removal, albeit with a clear reduction in GFP signals, suggesting that the GFP signal decrease may be the result of transcriptional silencing in our plasmid expression cassettes. Nonetheless, our results indicate that during asexual blood growth the plasmid containing the PvCEN11S2 region was stably maintained equally well in parasites with low copy number and the *P*. *yoelii* centromere-based plasmid.

### Transgenic parasites were able to retain plasmids with *P*. *vivax* centromere regions through the mosquito stages of the parasite life cycle

**Fig 4:**
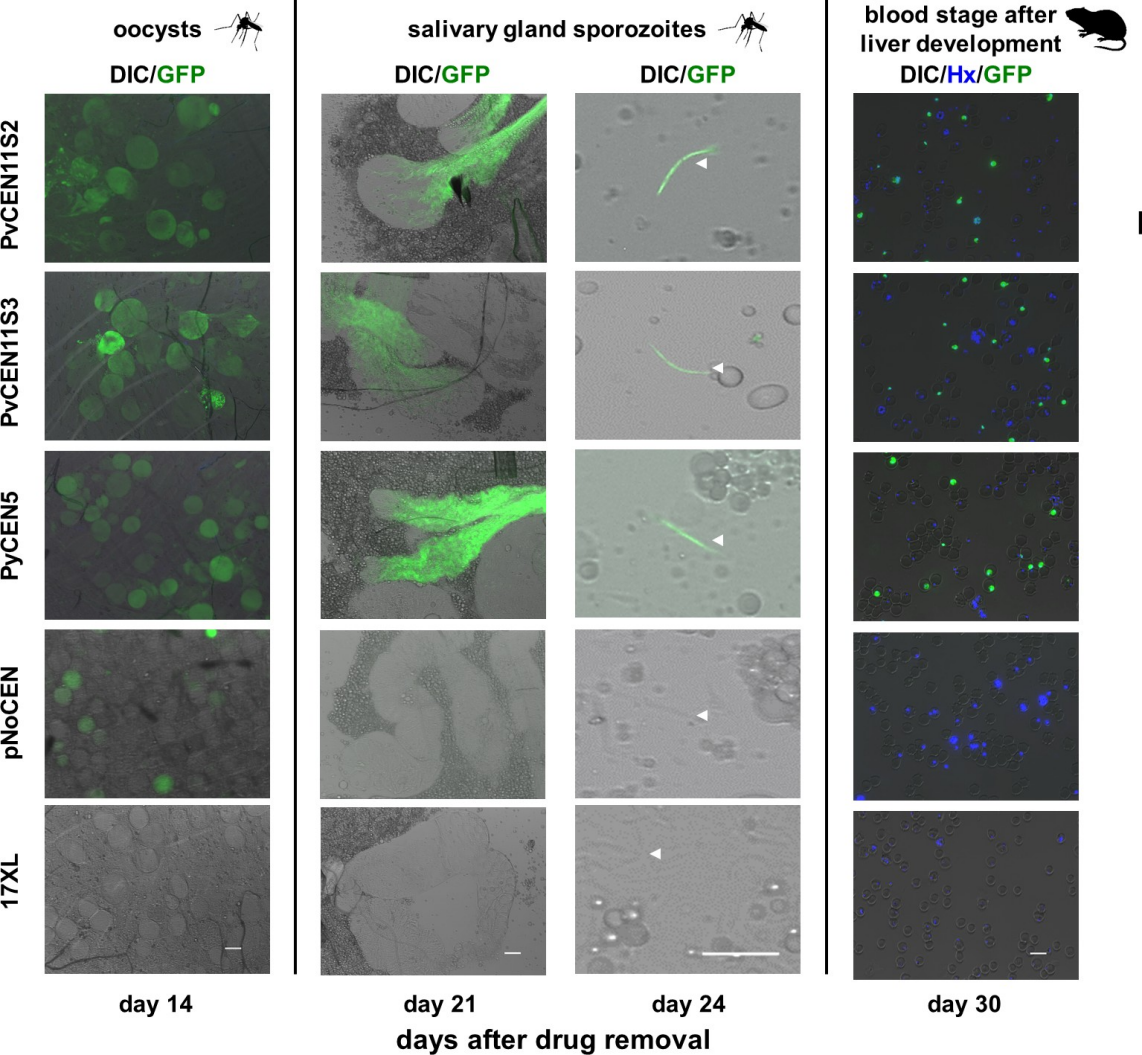
GFP signals from mosquito midgut oocysts, salivary gland sporozoites, and blood stages after liver stage development of transfected parasites. Forty mosquitoes were fed on day 3 after intravenous injection of 10^6^ infected red blood cells into mice for each group (transgenic parasites with PvCEN11S2-H86HD-TG (PvCEN11S2), PvCEN11S3-H86HD-TG (PvCEN11S3), PyCEN5-H86HD-TG (PyCEN5), or pNoCEN and the parental parasite 17XL) and were dissected to count the numbers of midgut oocysts and to observe GFP signals on day 11 after feeding (day 14 after pyrimethamine removal). Whole salivary glands were examined to detect GFP signals on day 18 (day 21 after pyrimethamine removal). Ten to fifteen remaining mosquitoes were dissected to collect salivary gland sporozoites on day 21 after feeding (day 24 after pyrimethamine removal). Sporozoites were counted and observed for GFP signals. All possible collected sporozoites were injected intravenously into mice and parasites that appeared in the blood on day 6 after inoculation (day 30 after pyrimethamine removal) were observed for a GFP signal. Parasite nuclei were stained with Hoechst (Hx). DIC, differential interference contrast image. Bar indicates 10 μm.

**Fig 5:**
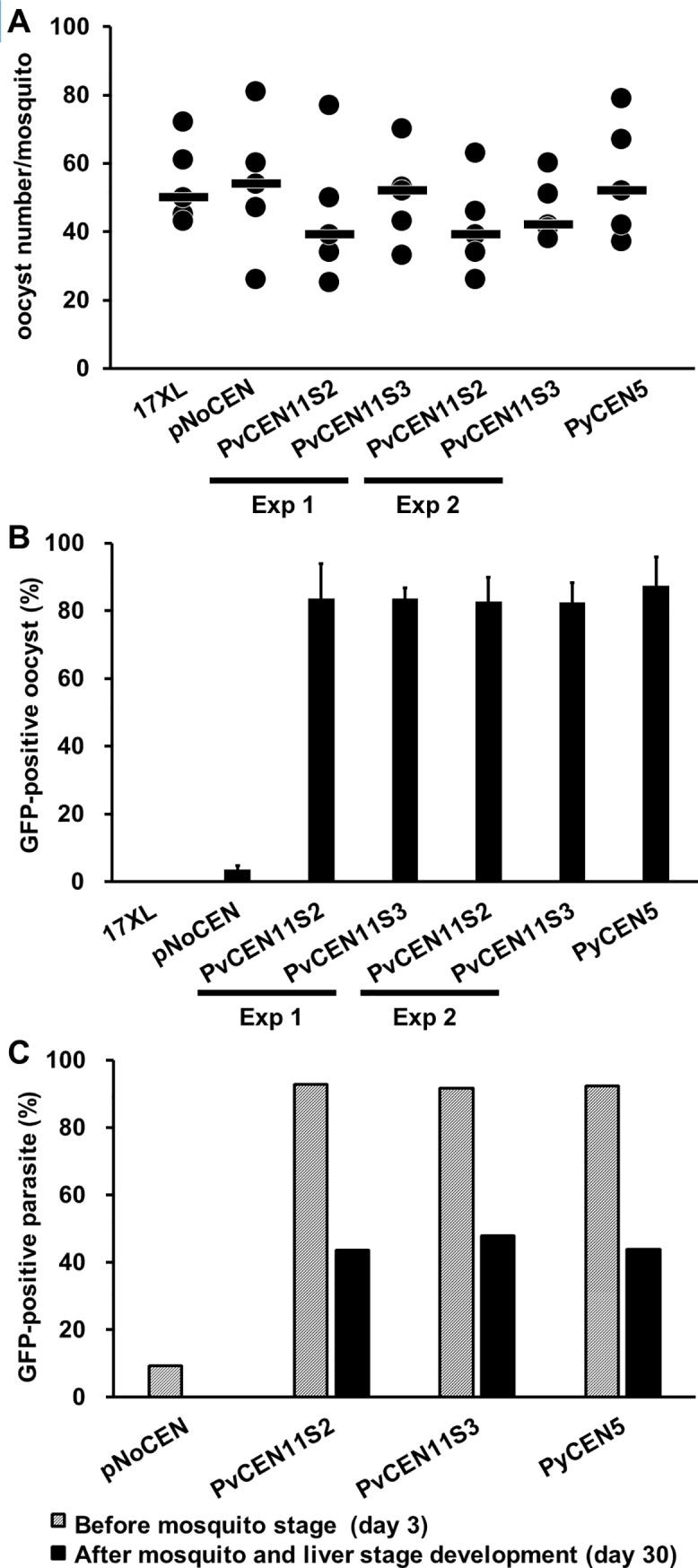
Percentage of GFP-positive oocysts and blood-stage parasites before and after mosquito and liver stage development. Oocyst numbers **(A)** and the percentage of GFP-positive oocysts **(B)** were examined for 5 mosquitoes on day 11 after feeding of mice infected with transgenic parasites with PvCEN11S2-H86HD-TG (PvCEN11S2), PvCEN11S3-H86HD-TG (PvCEN11S3), PyCEN5-H86HD-TG (PyCEN5), pNoCEN, and the parental parasite 17XL. Two independent experiments were performed for PvCEN11S2 and PvCEN11S3 transgenic parasites. **(A)** Black circles and bars indicate the median and distribution of oocyst numbers in each mosquito. **(C)** Percentage of GFP-positive blood-stage parasites were examined on the feeding day (day 3 after pyrimethamine removal) and after mosquito and liver stage development (day 30 after pyrimethamine removal).

We examined if *P*. *vivax* centromere-based plasmids were stably maintained in parasites through the mosquito stages. On day 11 after mosquito feeding (day 14 after PYR removal), the median oocyst numbers in mosquito midguts were 39 and 52 (1st experiment) or 39 and 42 (2nd experiment) for PvCEN11S2 and PvCEN11S3, respectively. In contrast, those for PyCEN5 and pNoCEN transgenic parasites and *P*. *yoelii* wild type parasite were 52, 54, and 50, respectively (Figs 4 and 5A). These results indicate that all parasite lines yielded sufficient numbers of oocysts. Greater than 80% of oocysts showed GFP signals in all transgenic parasite lines with centromere-based plasmids, whereas pNoCEN parasites showed only 4% (Figs 4 and 5B). Salivary gland sporozoites collected on day 18 after mosquito feeding (day 21 after PYR removal) showed GFP signals only in PvCEN11S2, PvCEN11S3, and PyCEN5 transgenic parasite lines. No GFP signal was seen in pNoCEN parasites and *P*. *yoelii* wild type parasites (Fig 4). Due to a limitation of mosquito numbers, we did not evaluate the percentage of GFP-positive sporozoites. On day 3 or 4 after intravenous inoculation of pooled sporozoites (6,000~10,500) into naïve mice, blood-stage parasites emerged in all lines. On day 6 after mouse inoculation (day 30 after PYR removal), at least 40% of the blood-stage parasites showed GFP signals in PvCEN11S2, PvCEN11S3, and PyCEN5 transgenic parasite lines, whereas pNoCEN parasites and *P*. *yoelii* wild type parasite did not show a GFP signal (Figs 4 and Fig 5C). We found that PvCEN11S3 parasites retained GFP positivity as efficiently as PvCEN11S2 and PyCEN5 after mosquito and liver stage development, and the pattern of plasmid copy numbers with or without PYR pressure during the blood-stages were clearly different (Fig 3). It is formally possible that the lesser or absent GFP signal in pNoCEN parasites in the mosquito stages is not due to a loss of the plasmid, but a lack of transcription under the *P*. *vivax* HSP86 promoter in the pNoCEN plasmid; however, because HSP86 is shown to be highly transcribed in the *P*. *vivax* sporozoite stage [28] and pNoCEN blood-stage parasites did not show a GFP signal after passing through mosquitoes, we considered that the lack of GFP signal was due to a loss of the plasmid. Nonetheless, these results indicated that plasmids with PvCEN11S2 and PvCEN11S3 were maintained throughout the mosquito and liver stages of the parasite life cycle as efficiently as the PyCEN5 plasmid.

### Evaluation of *P*. *vivax* promoters in blood and mosquito stages

**Fig 6:**
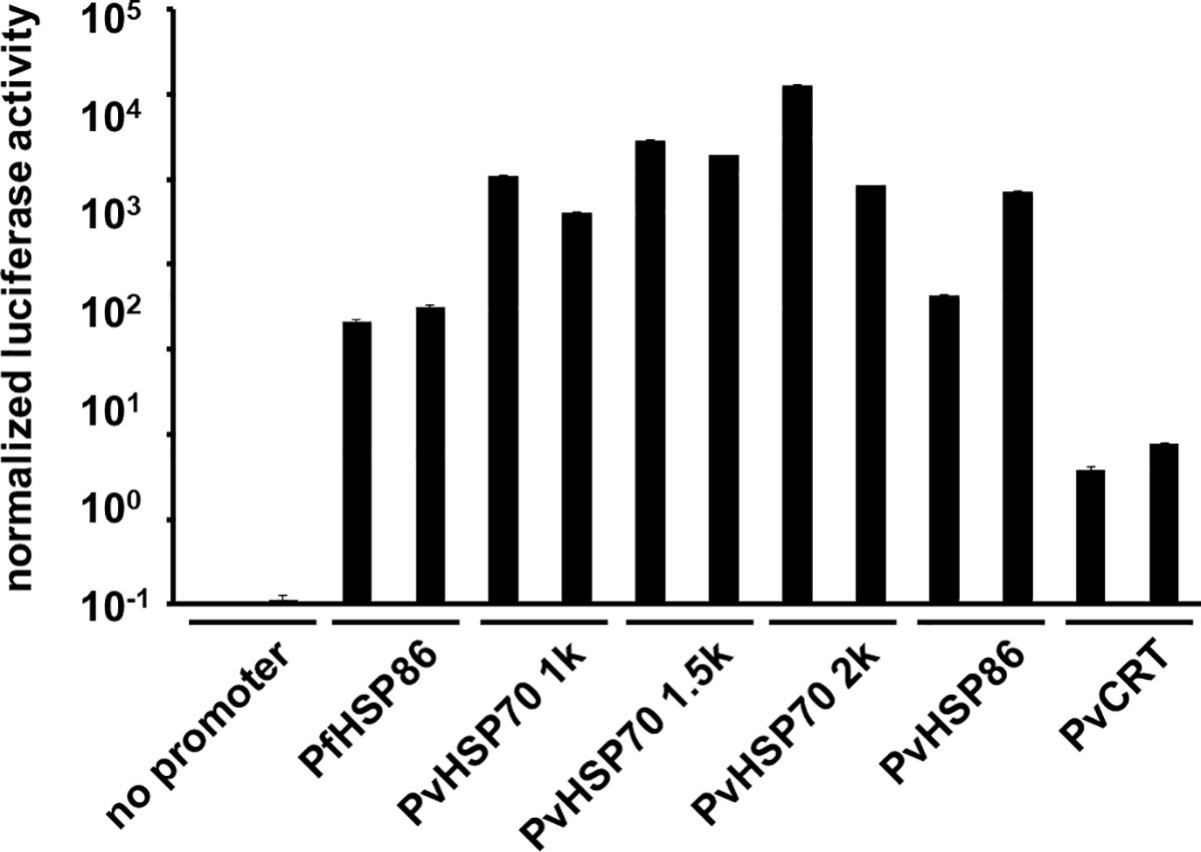
*P*. *vivax* promoter activities in *P*. *yoelii*. Firefly luciferase was expressed under a panel of *P*. *vivax* promoters. *P*. *falciparum* heat shock protein 86 promoter was used as a positive control, because this promoter has been shown to be active in *P*. *yoelii*. A plasmid without a promoter sequence upstream of the luciferase open reading frame was used as a negative control. Luciferase activities from purified schizont stage parasites were normalized by the plasmid copy number of each parasite quantitated by qPCR. Results from two independent experiments are shown. Mean and standard deviation from triplicate wells are plotted.

To expand the repertoire of validated *P*. *vivax* promoters, we cloned from *P*. *vivax* gDNA the 5' untranslated regions (UTR) of *hsp70*, *hsp86*, and *crt*. For *pvhsp70* 5' UTR, 3 fragments with different lengths (approximately 1, 1.5, and 2 kb) were evaluated. The *pfhsp86* promoter was used as a positive control. Two independent transgenic *P*. *yoelii* lines were generated for each plasmid and firefly luciferase activities were compared from purified schizont-stage parasites. Significantly higher luciferase activities were detected from all evaluated 5' UTR, with *hsp70* promoter as the strongest, followed by the *pvhsp86* and *pvcrt* promoters (Fig 6) by comparison with a negative control plasmid without a promoter.

**Fig 7:**
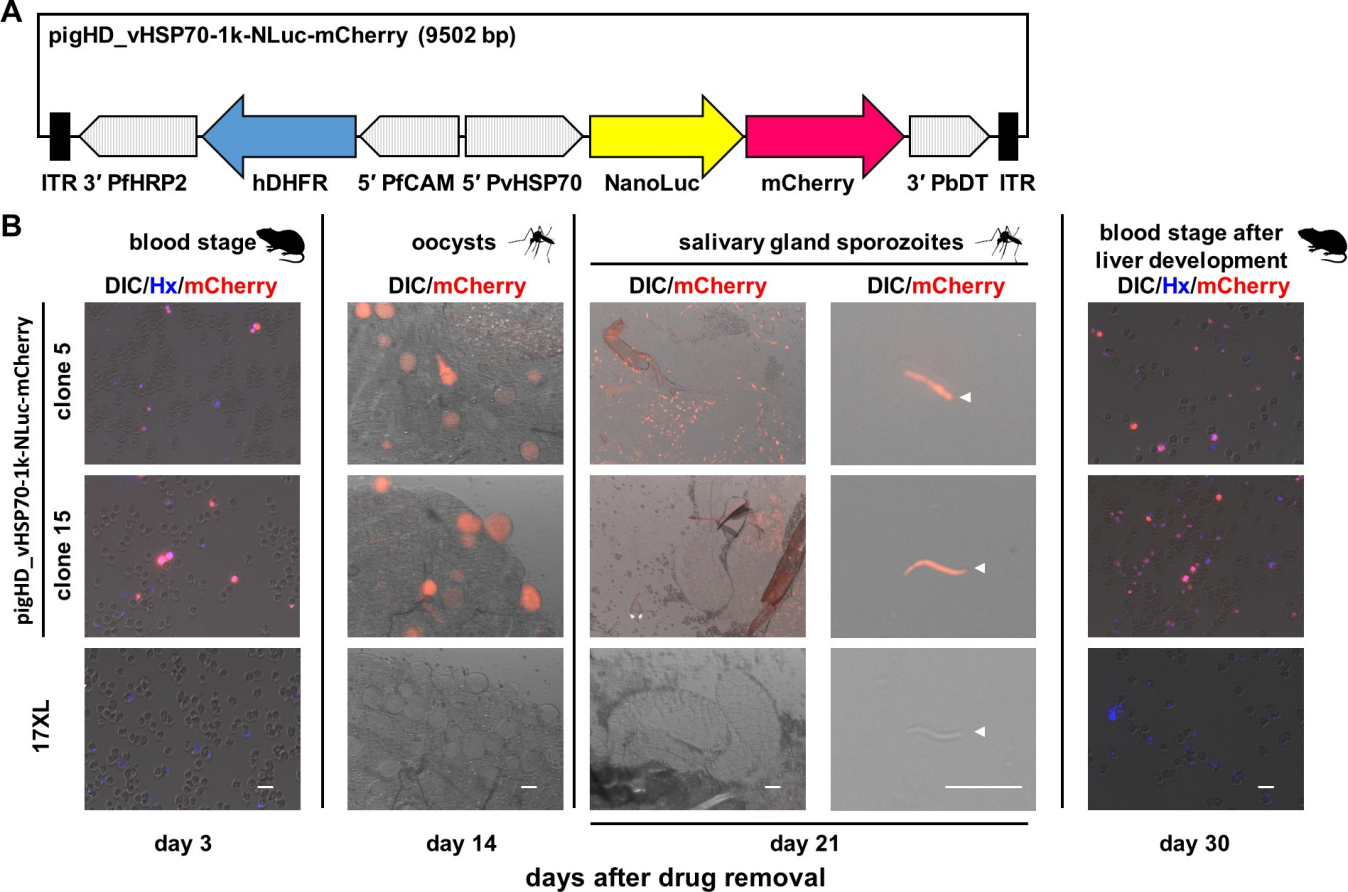
mCherry signals from blood-stage parasites, mosquito midgut oocysts, salivary gland sporozoites, and blood stages after liver stage development of transfected parasites with pigHD_vHSP70-1k-NLuc-mCherry. **(A)** Schematic image of pigHD_vHSP70-1k-NLuc-mCherry, a *piggyBac*-based plasmid expressing NanoLuc^®^ Luciferase-mCherry fusion protein under the *pvhsp70* promoter (~1 kb). 5' PvHSP70, the 5' untranslated region (UTR) of *P*. *vivax* heat shock protein 70; 3' PbDT, 3' UTR of *P*. *berghei* dihydrofolate reductase-thymidine kinase; 5' PfCAM, 5' UTR of *P*. *falciparum* calmodulin; hDHFR, human dihydrofolate reductase open reading frame; 3' PfHRP2, 3' UTR of *P*. *falciparum* histidine-rich protein 2; ITR, inverted terminal repeat sequences for the *piggyBac* transposon system. **(B)** Mice were infected with transgenic parasites with pigHD_vHSP70-1k-NLuc-mCherry or the parental parasite 17XL by intravenous injection of 10^6^ infected red blood cells and maintained without pyrimethamine. Three days later thin blood smears were made (day 3) and mosquitoes were fed. Mosquitoes were dissected on day 11 after feeding (day 14 after pyrimethamine removal) to count the number of the midgut oocysts and to observe mCherry signals. Whole salivary glands were examined to detect mCherry signals on day 18 (day 21 after pyrimethamine removal). The remaining mosquitoes were dissected to collect salivary gland sporozoites on day 24, which were then injected intravenously into mice. Parasites appeared in the blood on day 6 after inoculation (day 30 after pyrimethamine removal) and were observed for mCherry signals. Parasite nuclei were stained with Hoechst (Hx). DIC, differential interference contrast image. Bar indicates 10 μm.

**Fig 8:**
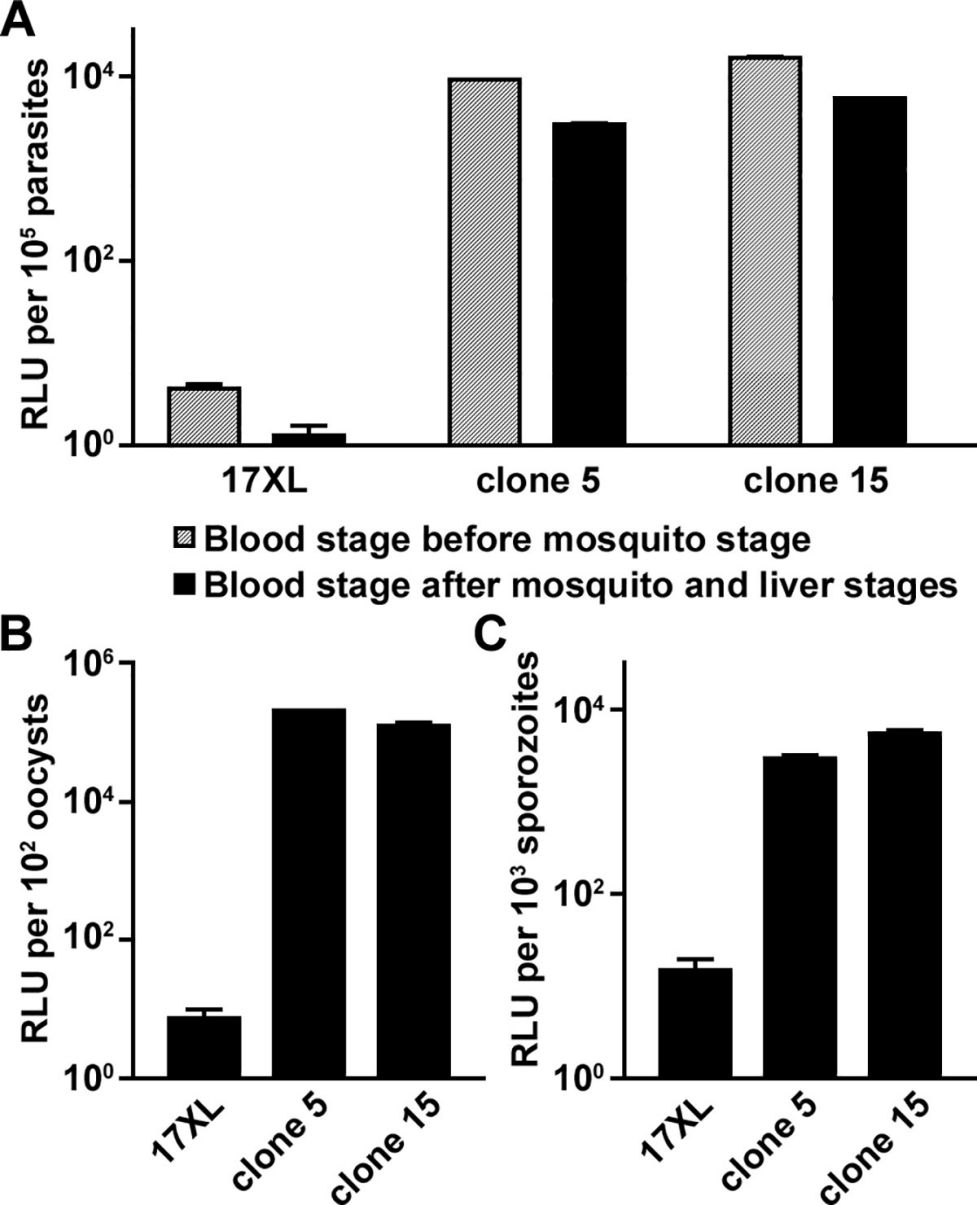
NanoLuc luciferase activities from various stages of transfected parasites with pigHD_vHSP70-1k-NLuc-mCherry. Clones 5 and 15 are cloned transfectants with pigHD_vHSP70-1k-NLuc-mCherry. *P*. *yoelii* 17XL was used as a negative control. Relative luciferase activities (relative light units, RLU) from 10^5^ blood-stage parasites before and after mosquito and liver stage development **(A)**, 10^2^ mosquito midgut oocysts **(B)**, or 10^3^ mosquito salivary gland sporozoites **(C)** are shown. Mean and standard deviations from triplicate wells are plotted.

An ~1 kb fragment of the *pvhsp70* 5' UTR was selected for further evaluation because in other *Plasmodium* species this promoter has been frequently used to express proteins throughout the parasite life cycle including mosquito and liver stages. A *piggyBac*-based plasmid expressing NanoLuc^®^ Luciferase-mCherry fusion protein under the *pvhsp70* promoter (~1 kb) was generated and transfected to *P*. *yoelii* with a helper pHTH plasmid [29]. From two transfectant clones obtained (clones 5 and 15), bright mCherry-positive parasites were observed at blood, oocyst, and salivary gland sporozoite stages (Fig 7). Luciferase activities were detected before and after mosquito and liver stage development, indicating that the expression from the plasmid-inserted region was stable (Fig 8A). Luciferase activity was also detected in oocyst (Fig 8B) and salivary gland sporozoite (Fig 8C) stages. These results indicate that the *pvhsp70* promoter was active throughout the mosquito stages.

## Conclusions

In this study, we cloned *P*. *vivax* centromere and promoter regions and confirmed their activities using *P*. *yoelii*. These experimentally validated components would serve as valuable tools for genetic manipulations to study *P*. *vivax*.

## Materials and methods

### Plasmid construction

*P*. *vivax* centromere sequences of chromosomes 5, 9, 11, and 13 (Genbank numbers CM000446.1, CM000450.1, CM000452.1, and CM000454.1, respectively) were PCR-amplified from *P*. *vivax* (SalI line) genomic DNA (gDNA) with primers PvCEN5.F and PvCEN5.R for PvCEN5 (2484 bp), PvCEN9.F and PvCEN9.R for PvCEN9 (2583 bp), PvCEN11.F and PvCEN11.R for PvCEN11 (2597 bp), and PvCEN13.F and PvCEN13.R for PvCEN13 (2645 bp). The *P*. *yoelii* centromere sequence of chromosome 5 (DQ054838.1) was PCR-amplified from *P*. *yoelii* 17XL line gDNA with primers PyCEN5.F and PyCEN5.R for PyCEN5 (1788 bp) [26]. Amplified PCR fragments were cloned into the pGEM-T easy plasmid (Promega) using the *E*. *coli* TOP10 strain (Thermo Fisher Scientific) to yield pGEM-PvCEN5, -9, -11, -13, and pGEM-PyCEN5, respectively. Two short versions of the chromosome 11 centromere region, including repeat sequence motif (white arrowhead in Fig 1), were amplified with primers PvCEN11.F3 and PvCEN11.R3 for PvCEN11S2 (1888 bp) or PvCEN11.F4 and PvCEN11.R4 for PvCEN11S3 (1535 bp) from pGEM-PvCEN11 and ligated into the pGEM-T easy plasmid, yielding pGEM-PvCEN11S2 and pGEM-PvCEN11S3, respectively. A DNA fragment containing Gateway *ccdB* R43 (Invitrogen, Carlsbad, CA) and *P*. *berghei* DHFR-TS 3' UTR (PbDT3U) was amplified from pCHD43(II) [30, 31] with primers SpeI-M13R and SacI-PbDT3U.R2 and ligated between the SpeI and SacI sites of pGEM-PvCEN11S2, -PvCEN11S3, and -PyCEN5, yielding pDST-PvCEN11S2, -PvCEN11S3, and -PyCEN5, respectively. The *P*. *vivax* heat shock protein 86 (HSP86; PVX_087950) promoter region was amplified from *P*. *vivax* gDNA with primers PvHSP86-5U.B4F and PvHSP86-5U.B1R and recombined with pDONR P4-P1r by the Gateway BP reaction (Invitrogen), yielding pENT41-PvHSP86-5U. A DNA fragment encoding human DHFR was amplified from pCHD43(II) with primers hDHFR.B1F and hDHFR.B2R and recombined with pDONR221 by the Gateway BP reaction, yielding pENT12-hDHFR. PvCEN11S2-H86HD-TG, PvCEN11S3-H86HD-TG, and PyCEN5-H86HD-TG were generated by the Gateway Multi LR reaction of one of the pDST plasmids, pENT41-PfHSP86-5U [30], pENT12-hDHFR, and pENT23-3TyGFP [32]. A DNA fragment containing Gateway *ccdB* R43 (Invitrogen) and *P*. *berghei* DHFR-TS 3' UTR was also amplified from pCHD43(II) with primers pCHD43(II).NrR1 and PbDT3U.R2 and cloned into pGEM-T easy, yielding pGEM-T-*ccdB*43-PbDT3U, which was further recombined with pENT41-PvHSP86, pENT12-hDHFR, and pENT23-3TyGFP, yielding pNoCEN.

Plasmids expressing luciferase were generated as follows. DNA fragments containing the 5' UTR of *P*. *vivax hsp70* (PVX_089425) and *crt* (PVX_087980) were amplified with primers PvHSP70-5U2.B4F5 and PvHSP70-5U3.B1R1, PvHSP70-5U2.B4F4 and PvHSP70-5U3.B1R1, PvHSP70-5U2.B4F3 and PvHSP70-5U3.B1R1, and PvCRT-5U.B4F and PvCRT-5U.B1R to generate pENT41-PvHSP70-5U-1k, pENT41-PvHSP70-5U-1.5k, pENT41-PvHSP70-5U-2k, and pENT41-PvCRT-5U, respectively. pENT12-Luc was generated by BP reaction of a DNA fragment PCR-amplified from pHDEF1-*luc* [33] with primers Luc.B1F and Luc.B2R and pDONR221. pENT23-2Myc was generated by BP reaction of the hybridized oligonucleotides (2Myc-2.B2F and 2Myc-2.B3R) and pDONR P2R-P3. The above pENT41 plasmids and pENT41-PvHSP86-5U were subjected to the MultiSite LR reaction with pENT12-Luc, pENT23-2Myc, and pCHD43(II). pCHD-PfHSP86-Luc-TG was made with pENT41-PfHSP86-5U, pENT12-Luc, pENT23-3TyGFP, and pCHD43(II). A control plasmid without a promoter region (pCHD-Luc-TG) was generated by removing the promoter region using primers Luc.F5 and CHD.R3.

pENT12-NLuc was generated using a DNA fragment encoding NanoLuc^®^ Luciferase amplified from pNL1.1 [Nluc] vector (Promega) with primers Nluc.B1F and Nluc.B2R (S1 Table).

The *piggyBac*-based Multisite Gateway destination plasmid (pigHD_R43-3U) was generated from pXL-BacII-DHFR, by inserting a linker (StSmNhE5.ApaF and StSmNhE5.ApaR) into an ApaI site, a DNA fragment containing PbDT3U amplified from pHH1 [34] with primers PbDT3U.F3 and PbDT3U.R3 to a SmaI site, then a DNA fragment containing *ccdB* R43 amplified from pCHD43(II) with primers M13F and M13R into a StuI site. pigHD_vHSP70-1k-NLuc-mCherry was generated from pENT41-PvHSP70-5U-1k, pENT12-NLuc, pENT23-mCherry, and pigHD_R43-3U plasmids.

### Malaria parasites, mice, and mosquitoes

The *P*. *yoelii* 17XL line was maintained using 6 to 8 week old female ICR mice (SLC Inc., Shizuoka, Japan) in Nagasaki University. Animal experiments conducted in this study were approved by the Animal Care and Use Committee of Nagasaki University (Permit number 1403031120–9) and mice were treated in accordance with the Act on Welfare and Management of Animals, Japan. Mice were kept in a temperature-controlled (20–26°C) room under a 12-hour light/dark cycle and monitored at least twice a week. Availability of food and water was also monitored at least twice a week. Malaria parasites were intravenously inoculated to mice anesthetized with isoflurane. Euthanization of mice was done by deep anesthesia with 2–5% isoflurane followed by cervical dislocation.

Forty *Anopheles stephensi* mosquitoes for each group were fed on day 3 after intravenous injection of 10^6^ parasite-infected RBCs into mice. Mosquitoes were dissected to observe midgut oocysts on day 11 after feeding (day 14 after PYR removal) and salivary gland sporozoites on day 18 (day 21 after PYR removal). To collect sporozoites for injection into mice, salivary glands were gently crushed in sterile phosphate buffered saline (PBS) using a plastic homogenizer and sporozoites were harvested from the supernatant after a brief low-speed centrifugation. Collected sporozoites were injected into mice intravenously.

### Generation of transgenic parasites

*P*. *yoelii* schizont-enriched fractions were collected from mouse blood by differential centrifugation on a 50% Histodenz ^TM^ (Sigma-Aldrich, St. Louis, MO) solution and 20 μg of each plasmid was electroporated to 5 × 10^7^ enriched schizonts using the Nucleofector^™^ 2b device (Lonza Japan) with Human T cell solution under program U-33 [35]. Transfected parasites were intravenously injected into 8-week old BALB/c female mice and transfectants were selected by oral administration of PYR and cloned by limiting dilution using mice. For pigHD_vHSP70-1k-NLuc-mCherry plasmid, 20 μg of pHTH plasmid was also transfected [29].

### Live imaging of the parasites

Thin smears of parasite-infected blood, spots of purified sporozoites, and oocyst-attached mosquito midguts were prepared on glass slides, stained with Hoechst 33342, and fluorescent and differential interference contrast (DIC) images were captured using an AxioCam MRm CCD camera (Carl Zeiss, Germany) fixed to an Axio imager Z2 fluorescent microscope with a Plan-Apochromat 100 ×/1.4 oil immersion lens (Carl Zeiss) and Axiovision software (Carl Zeiss). Obtained images were processed using Zen lite 2011 software (Carl Zeiss) and Adobe Photoshop CS (Adobe Systems Inc., San José, CA).

The segregation efficiency of the plasmid per nuclear division was calculated using the following equation:

$$segregation\;efficiency=100\times {\left(Pgfp/100\right)}^{1/n}$$,

where P_gfp_ is the percentage of GFP-positive parasites after PYR removal and n is the number of nuclear divisions. In this study, we assumed that 3.5 rounds of nuclear division (equivalent to the generation of 11 daughter parasites) occurred during one blood-stage schizogony of 18 hours.

### Quantitative polymerase chain reaction (qPCR)

Genomic DNA was purified from the mixed stage parasites collected from mice using the QiaAmp DNA Mini Kit (QIAGEN, Germany). qPCR was conducted to quantitate the plasmids (targeting GFP gene with primers GFP.rtF1 and GFP.rtR1) and parasite genome (targeting met-tRNA ligase gene (PY17X_0519800) with primers PyMet-tRNA.rtF1 and PyMet-tRNA.rtR1) with Power SYBR^™^ Green PCR Master Mix (Thermo Fisher Scientific) using the 7500 Real-Time PCR system (Applied Biosystems, Foster City, CA). Copy number was estimated by using standard curves generated with serially diluted plasmids containing target PCR product sequences. Values for the GFP gene were normalized to those for the met-tRNA ligase gene and values from three independent experiments were assessed. A one-way analysis of variance (ANOVA) test and Tukey's post-hoc multiple comparisons tests were performed using PRISM (GraphPad Software, San Diego, CA) to analyze the difference between the plasmid copy number per parasite genome.

### Luciferase assay

Purified schizont stage parasites were adjusted to 10^8^ parasites/assay and firefly luciferase assays were performed using the ONE-Glo^TM^ Luciferase system (Promega) according to the manufacturer's instructions. NanoLuc luciferase activity was measured by the Nano-Glo® Luciferase Assay system (Promega) according to the manufacturer's instructions. Luciferase signals were immediately measured with a plate reader (ARVO MX 1420; PerkinElmer) for 15 sec. Plasmid copy numbers for each sample were estimated by targeting hDHFR with primers hDHFR.rtF and hDHFR.rtR as described above, and used to normalize the luciferase activities.

## Supporting information

S1 Table Oligonucleotide primers used in this study. (PDF)

S1 Fig Maximum parasite growth rate per day and number of merozoite per schizont. **(A)** Maximum parasite growth rate per day. Although pNoCEN parasites showed lower value compared to the others, no significant difference was detected by one-way ANOVA test. **(B)** Number of merozoite per schizont were examined for 20 schizonts at day 3. No significant difference was detected by one-way ANOVA test.
(TIF)
