# Supplementary material for: Validation of Plasmodium vivax centromere and promoter activities using Plasmodium yoelii
Source: PLoS One. 2019 Dec 20;14(12):e0226884. doi: 10.1371/journal.pone.0226884 (PMC6924662; doi:10.1371/journal.pone.0226884)
Supplement: S1 Table — (PDF) [file pone.0226884.s001.pdf]

S1 Table. Oligonucleotide primers used in this study.

**plasmid**	**primer**	**sequence**
PvCEN5	PvCEN5.F	TGTCGAAAATTTTATAATCGTTAAAGAC
	PvCEN5.R	TAGTCTCATAAATGATCGATATTTCAC
PvCEN9	PvCEN9.F	GGAAAAACATGAAACACAATTTACATG
	PvCEN9.R	CATAACAAAACTTAAACATGCTTTGTC
PvCEN11	PvCEN11.F	ATTTTAAAGGAACTGTAGCATGTTAAC
	PvCEN11.R	TCACATAATTAACAACAGCAAAATCAG
PvCEN13	PvCEN13.F	TTGAAAGATGTATGATCCTGTATGAC
	PvCEN13.R	GGAATTCTCTGAATATGGCTAATATG
PyCEN5	PyCEN5.F	TAGACAAACAAAATGATTATGATACAAATAC
	PyCEN5.R	TTATAGGGTTATATTTTATAAGAAATAAGG
PvCEN11S2	PvCEN11.F3	AATATGACAATTTAATTAATTCACAAAGTAATATAAC
	PvCEN11.R3	TATAAAATAATTTTAATATATTGTACTTAACAAAAACG
PvCEN11S3	PvCEN11.F4	AATATTATTTTAGTTATTTTTAATAATAAATAATAAAAGG
	PvCEN11.R4	AAATTATGTTAAATAAAAAGAAATATTAACTTATAAAAG
To amplify a DNA fragment containing Gateway *ccdB* R43 and *P. berghei* DHFR-TS 3' UTR		
	SpeI-M13R	GACTACTAGTCAGGAAACAGCTATGACCATG
	SacI-PbDT3U.R2	TGACGAGCTCCCCTGAAGAAGAAAAGTCCG
pENT41-PvHSP86-5U (~1.2 kb)		
	PvHSP86-5U.B4F	ggggACAACTTTGTATAGAAAAGTTGTGCATGTGCGAAACGACACAGC
	PvHSP86-5U.B1R	ggggACTGCTTTTTTGTACAAACTTGTTTGCTTAGCGGGGGGGTAC
pENT12-hDHFR		
	hDHFR.B1F	ggggACAAGTTTGTACAAAAAAGCAGGCTAAAAAAATGCATGGTTCGCTAAACTGC
	hDHFR.B2R	ggggACCACTTTGTACAAGAAAGCTGGGTAATCATTCTTCTCATATACTTC
pGEM-T-*ccdB*43-PbDT3U		
	pCHD43(II).NrR1	CCCGGCGCCGGGAATACTCAAGCTGCGGCCC
	PbDT3U.R2	CCCTGAAGAAGAAAAGTCCG

* Gateway *attB* sites are underlined

S1 Table. Oligonucleotide primers used in this study (cont').

plasmid	primer	sequence
pENT41-PvHSP70-5U-2k		
	PvHSP70-5U2.B4F3	ggggACAACTTTGTATAGAAAAGTTGAAAATGGCTTCCTCACTGCGATG
	PvHSP70-5U3.B1R1	ggggACTGCTTTTTTGTACAAACTTGTTTGAGATTAGCGATTTAAAGG
pENT41-PvHSP70-5U-1.5k		
	PvHSP70-5U2.B4F4	ggggACAACTTTGTATAGAAAAGTTGACTGCCATATGCATAAAAAGGA
pENT41-PvHSP70-5U-1k		
	PvHSP70-5U2.B4F5	ggggACAACTTTGTATAGAAAAGTTGTTTCGCGGAAAAACACACGCAT
pENT41-PvCRT-5U		
	PvCRT-5U.B4F	ggggACAACTTTGTATAGAAAAGTTGTTAGGCCATCGCACAAAGGAATG
	PvCRT-5U.B1R	ggggACTGCTTTTTTGTACAAACTTGTGTGCGTTAGGGGTAGCGGTG
pENT12-Luc		
	Luc.B1F	ggggACAAGTTTGTACAAAAAAGCAGGCTAAAATGGAAGACGCCAAAAACATAA
	Luc.B2R	ggggACCACTTTGTACAAGAAAGCTGGGTACAATTTGGACTTTCCGCC
pENT23-2Myc		
	2Myc-2.B2F	ggggACAGCTTTCTTGTACAAAGTGGGATCCATGGGGCCCGAACAAAAACTCATCTCAGAAGAG<br>GATCTGGAACAGAAGTTAATAAGTGAGGAAGACTTATAACAACTTTATTATACAAAGTTGTcccc
	2Myc-2.B3R	ggggACAACTTTGTATAATAAAGTTGTTATAAGTCTTCCTCACTTATTAACTTCTGTTCCAGATCC<br>TCTTCTGAGATGAGTTTTTGTTCGGGCCCCATGGATCCCACTTTGTACAAGAAAGCTGTcccc
pCHD-Luc-TG		
	Luc.F5	GCTAAAATGGAAGACGCCAAAAACAT
	CHD.R3	ATAGCTTGGCGTAATCATGGTCATAG.
pigHD_R43-3U		
	StSmNhE5.ApaF	AGGCCTCCCGGGCTAGCGATATCGGCC
	StSmNhE5.ApaR	GATATCGCTAGCCCGGGAGGCCTGGCC
	PbDT3U.F3	GGATATGGCAGCTTAATGTTCG
	PbDT3U.R3	CCCTGAAGAAGAAAAGTCCG

* Gateway *attB* sites are underlined

S1 Table. Oligonucleotide primers used in this study (cont').

plasmid	primer	sequence
pENT12-NLuc		
	Nluc.B1F	ggggACAAGTTTGTACAAAAAAGCAGGCTAAAAAAATGGTCTTCACACTCGA
	Nluc.B2R	ggggACCACTTTGTACAAGAAAGCTGGGTACGCCAGAATGCGTTC
pENT23-mCherry		
	B2-BamHI-mCherry-F	ggggCAGCTTTCTTGTACAAAGTGGGTGGATCCATGGTGAGCAAGGGCGAGG
	B3-SX-mCherry(ST)-Rv2	ggggCAACTTTGTATAATAAAGTTGCCCGGGCTCGAGTTACTTGTACAGCTCGTCCATGCC
qPCR to target the plasmid GFP gene		
	GFP.rtF1	TGTTCCATGGCCAACACTTGTC
	GFP.rtR2	ACGGGAACTACAAGACACGTGC
qPCR to target *P. yoelii* met-tRNA gene		
	PyMet-tRNA.rtF1	AAAAATGTCGAAATCTTTAAAAAACGTG
	PyMet-tRNA.rtR1	TCAGCTAAATCAGAGTTACACATATC
qPCR to target the plasmid hDHFR gene		
	hDHFR.rtF	GGTCTGGATAGTTGGTGGTGGCA
	hDHFR.rtR	AACACCTGGGGTATTCTGGCA

* Gateway *attB* sites are underlined
