# Supplementary figures and images for: Validation of Plasmodium vivax centromere and promoter activities using Plasmodium yoelii
Source: PLoS One. 2019 Dec 20;14(12):e0226884. doi: 10.1371/journal.pone.0226884 (PMC6924662; doi:10.1371/journal.pone.0226884)

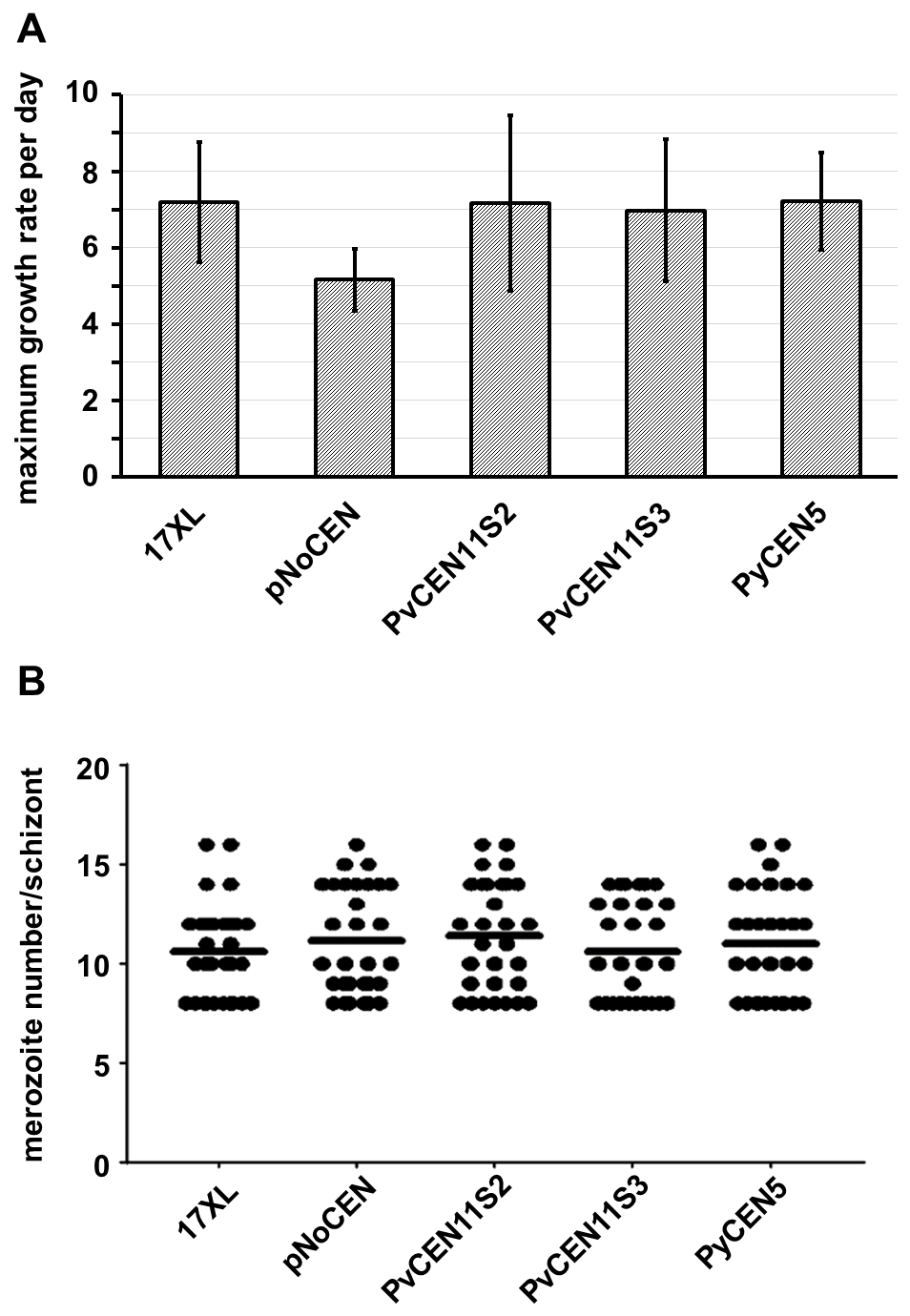

Supplement: S1 Fig — (A) Maximum parasite growth rate per day. Although pNoCEN parasites showed lower value compared to the others, no significant difference was detected by one-way ANOVA test. (B) Number of merozoite per schizont were examined for 20 schizonts at day 3. No significant difference was detected by one-way ANOVA test. (TIF) [file pone.0226884.s002.tif]
